# The cross talk between type II diabetic microenvironment and the regenerative capacities of human adipose tissue-derived pericytes: a promising cell therapy

**DOI:** 10.1186/s13287-024-03643-1

**Published:** 2024-02-08

**Authors:** Toka A. Ahmed, Sara M. Ahmed, Hoda Elkhenany, Mohamed A. El-Desouky, Sameh Magdeldin, Aya Osama, Ali Mostafa Anwar, Ihab K. Mohamed, Mohamed Essameldin Abdelgawad, Demiana H. Hanna, Nagwa El-Badri

**Affiliations:** 1 Center of Excellence for Stem Cells and Regenerative Medicine (CESC), Zewail City of Science and Technology, October Gardens, 6th of October City, Giza 12582 Egypt; 2 Egypt Center for Research and Regenerative Medicine (ECRRM), Cairo, Egypt; 3 Department of Surgery, Faculty of Veterinary Medicine, Alexandria University, Alexandria, 22785 Egypt; 4 Department of Chemistry, Faculty of Science, Cairo University, Giza, 12613 Egypt; 5 Proteomics and Metabolomics Research Program, Basic Research Department, Children’s Cancer Hospital, Cairo, 57357 Egypt; 6 Department of Zoology, Faculty of Science, Ain Shams University, Cairo, Egypt; 7 Biochemistry and Molecular Biotechnology Division, Chemistry Department, Faculty of Science, Innovative Cellular Microenvironment Optimization Platform (ICMOP), Precision Therapy Unit, Helwan University, Cairo, Egypt; 8 The Egyptian Network of Bioinformatics “BioNetMasr”, Cairo, Egypt; 9 Department of Physiology, Faculty of Veterinary Medicine, Suez Canal University, Ismailia, Egypt

**Keywords:** Type 2 diabetes mellitus, Angiogenesis, Vascular complications, Cellular therapy, Pericytes

## Abstract

**Background:**

Pericytes (PCs) are multipotent contractile cells that wrap around the endothelial cells (ECs) to maintain the blood vessel's functionality and integrity. The hyperglycemia associated with Type 2 diabetes mellitus (T2DM) was shown to impair the function of PCs and increase the risk of diabetes complications. In this study, we aimed to investigate the deleterious effect of the diabetic microenvironment on the regenerative capacities of human PCs.

**Methods:**

PCs isolated from human adipose tissue were cultured in the presence or absence of serum collected from diabetic patients. The functionality of PCs was analyzed after 6, 14, and 30 days.

**Results:**

Microscopic examination of PCs cultured in DS (DS-PCs) showed increased aggregate formation and altered surface topography with hyperbolic invaginations. Compared to PCs cultured in normal serum (NS-PCs), DS-PCs showed more fragmented mitochondria and thicker nuclear membrane. DS caused impaired angiogenic differentiation of PCs as confirmed by tube formation, decreased *VEGF-A* and *IGF-1* gene expression, upregulated TSP1, PF4, actin-related protein 2/3 complex, and downregulated COL21A1 protein expression. These cells suffered more pronounced apoptosis and showed higher expression of Clic4, apoptosis facilitator BCl-2-like protein, serine/threonine protein phosphatase, and caspase-7 proteins. DS-PCs showed dysregulated DNA repair genes *CDKN1A, SIRT1, XRCC5 TERF2*, and upregulation of the pro-inflammatory genes *ICAM1, IL-6,* and *TNF-α*. Further, DS-treated cells also showed disruption in the expression of the focal adhesion and binding proteins TSP1, TGF-β, fibronectin, and PCDH7. Interestingly, DS-PCs showed resistance mechanisms upon exposure to diabetic microenvironment by maintaining the intracellular reactive oxygen species (ROS) level and upregulation of extracellular matrix (ECM) organizing proteins as vinculin, IQGAP1, and tubulin beta chain.

**Conclusion:**

These data showed that the diabetic microenvironment exert a deleterious effect on the regenerative capacities of human adipose tissue-derived PCs, and may thus have possible implications on the vascular complications of T2DM. Nevertheless, PCs have shown remarkable protective mechanisms when initially exposed to DS and thus they could provide a promising cellular therapy for T2DM.

**Graphical abstract:**

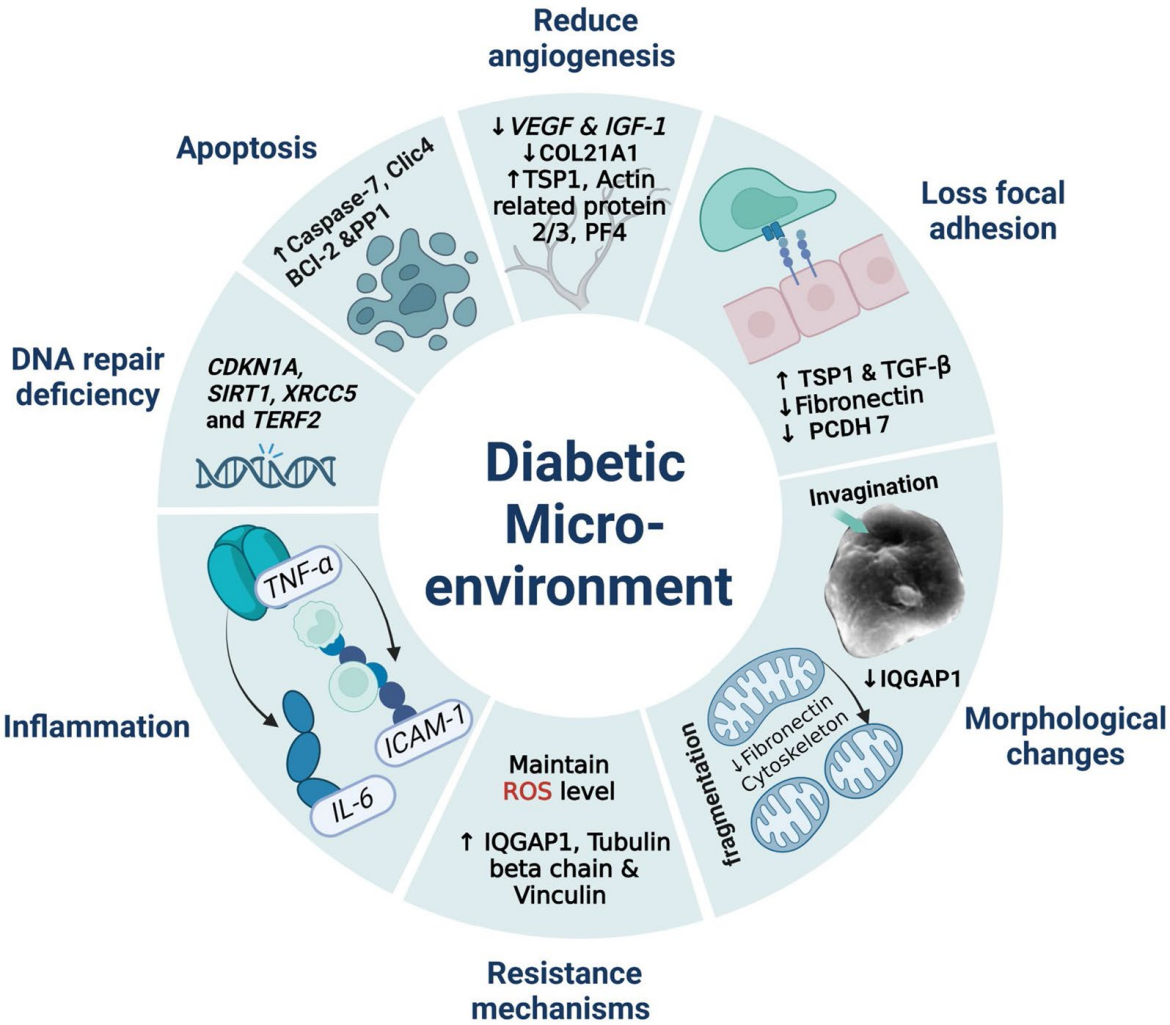

**Supplementary Information:**

The online version contains supplementary material available at 10.1186/s13287-024-03643-1.

## Introduction

Diabetes mellitus (DM) is one of the most common metabolic disorders and a leading cause of morbidity and mortality worldwide [1]. According to the World Health Organization, the prevalence of DM will be doubled by 2030 [2] and more than 960 million people will suffer from DM by 2045 [3]. DM results either from insulin resistance or defect in insulin production [4]. T2DM is the most common type with 90 to 95% prevalence among diabetic patients [5]. T2DM is characterized by progressive insulin resistance, increasing the body’s demand for insulin to maintain the blood glucose level. Inability to secrete a sufficient amount of insulin leads to progressive hyperglycemia and the development of DM. Sustained hyperglycemia leads to several metabolic abnormalities and can lead to various pathologies in the vascular system, kidneys, lungs, peripheral nerves, and eyes [6]. It was previously reported that the diabetic microenvironment impacts several cells including adult stem cells. PCs are type of adult stem cells described more than a 100 years ago as perivascular cells that wrap blood capillaries. The main role of PCs is to interact with ECs via different pathways to maintain the blood vessels' integrity throughout the body [7]. Several reports suggested a deleterious effect for the diabetic microenvironment on the physiological functions of PCs [3]. In addition, it has been reported that the long-lasting frequent hyperglycemic conditions observed in diabetic patients is associated with different microvascular complications such as diabetic retinopathy, neuropathy, and nephropathy. Diabetic retinopathy is characterized by different abnormalities including PC loss, increased vascular permeability, and increased thickness of the basement membrane [8]. The hyperglycemic microenvironment in DM was reported to induce apoptosis of retinal PCs [9]. The ECs-PCs crosstalk is essential for maintaining the microvasculature functionality, integrity, and hemostasis and can be disrupted in diabetic patients, which in turn affects the PC coverage and functionality [3, 8]. The loss of PCs from microvessels under diabetic conditions was first reported in the 1960s [10] and later in the skeletal muscles in 1985 [11]. PC's loss was shown to be associated with different complications of DM such as diabetic neuropathy, retinopathy, and nephropathy [3].

The cross talk between PCs, as a critical component of the microvascular system, and T2DM microenvironment was reported to affect various functions. T2DM has been reported to impair the physiological functionality and number of PCs in different tissues, which in turn promoted the vascular complications in diabetic patients [3, 12]. Dysfunction of PCs in the kidneys, retina, skeletal muscle, and pancreas has been reported in T2DM [3]. In animal studies, selective ablation of murine pancreatic islet PCs caused impaired glucose tolerance and increased fasting blood glucose level. Despite normal beta-cell number and islets morphology, transgenic mice with depleted islets PCs showed decreased level of insulin secretion and displayed markers of immature beta cells, suggesting that PCs are essential for optimal beta-cell functionality and maturity [13]. Patients suffering from T2DM had decreased skeletal muscles PCs coverage, and their PCs showed ultrastructural abnormalities. These PCs exhibited less myogenic differentiation potential and decreased capacity to support in vitro EC tube formation [14]. The regenerative potential of PCs has been previously reported [15], as they were shown to differentiate into adipocytes, osteocytes, myocytes, chondrocytes, and endothelial like cells [16, 17]. PCs thus present a new promising cellular therapy approach for different vascular diseases as they have not only maintained the integrity of blood vessels but also have an important role in immunomodulation, angiogenesis migration, and blood flow regulation [18, 19]. PCs have been reported as a promising cellular therapy for ischemic foot ulcer and enhancing the wound healing in diabetic mouse models. PCs were shown to delay human umbilical vein endothelial cell (HUVEC) senescence, which suggested that they may decrease the development of ischemic foot ulcer in diabetic patients [18]. It is worth mentioning that PCs have been reported to withstand harsh conditions such as ischemic environment. Indeed, low oxygen level allows them to perform their pro-angiogenic functions, secrete angiogenic factors, and secure the oxygen level to more energy demanding cells [19–23]. Additionally, it has been reported that PCs accommodate low oxygen level that they secrete the non-insulin-dependent glucose transporter, GULT-1, and utilize glycolysis instead of mitochondria oxidative phosphorylation [24–26]. Furthermore, PCs showed a modest dependence on mitochondrial ATP production despite their mitochondrial bioenergetics reserve capacity, suggesting that they are working only at a fraction of their total capacity [25]. This metabolic profile and resistance mechanisms could explain the PCs’ ability to withstand in vitro hypoxic conditions and survive when transplanted into rodent models of myocardial and peripheral ischemia [21, 22, 25, 27–29]. PCs have thus displayed a sufficient degree of metabolic flexibility and resistance within both ischemic and preferable metabolic conditions, which could enhance their therapeutic activity [19–22, 27, 28, 30]. Human serum is an aqueous solution, rich in a wide array of components, including growth factors, hormones, enzymes, lipoproteins, electrolytes, nutrients, and small organic molecules [31–33]. Serum was thus used as a physiologically relevant representative for the microenvironment [34, 35]. Serum factors were reported to affect the structure and functionality of several types of stem cells including cardiac, placental, umbilical cord-derived mesenchymal stem cells, and PCs [36–38]. In this study, we investigated the deleterious effect of the diabetic microenvironment on the regenerative capacities of human adipose tissue-derived PCs in order to determine target specific signaling pathways against diabetic complications. Moreover, we investigated the protective mechanism(s) of PCs upon exposure to DS, to determine PCs application as a promising cellular therapy for T2DM.

## Material and methods

### Normal and diabetic blood collection

Normal and diabetic blood samples were collected according to the protocol approved by the Ethical Committee of Kasr Alainy Faculty of Medicine, Cairo University (#R-8–2021), and after obtaining the patients’ informed consent. Blood samples were collected from fifteen T2DM patients who satisfied the American Diabetes Association criteria. All patients had no history of diabetic complications and received insulin therapy alone (*n* = 3) or insulin and Metformin (*n* = 12). Exclusion criteria included patients receiving hormone replacement therapy or thiazolidinedione, and patients with proteinuria, thyroid disease, or other known medical conditions. Pregnant females, alcoholics, and smokers have been excluded as well. The non-diabetic normal serum was collected from 15 non-diabetic normoglycemic, gender and age-matched healthy volunteers who were not receiving medications.

### Serum isolation from human blood samples

Venous blood samples from healthy and diabetic volunteers were collected into vacutainers containing no additives (BD Biosciences, Franklin Lakes, NJ, USA). Blood samples were allowed to clot before centrifugation at 2000 × g for 10 min at 4 °C. Afterward, all serum samples were pooled, filtered using a 0.2 µl syringe filter (Sigma-Aldrich, USA) and stored at − 20 °C.

### Pericyte culture

**Fig. 1:**
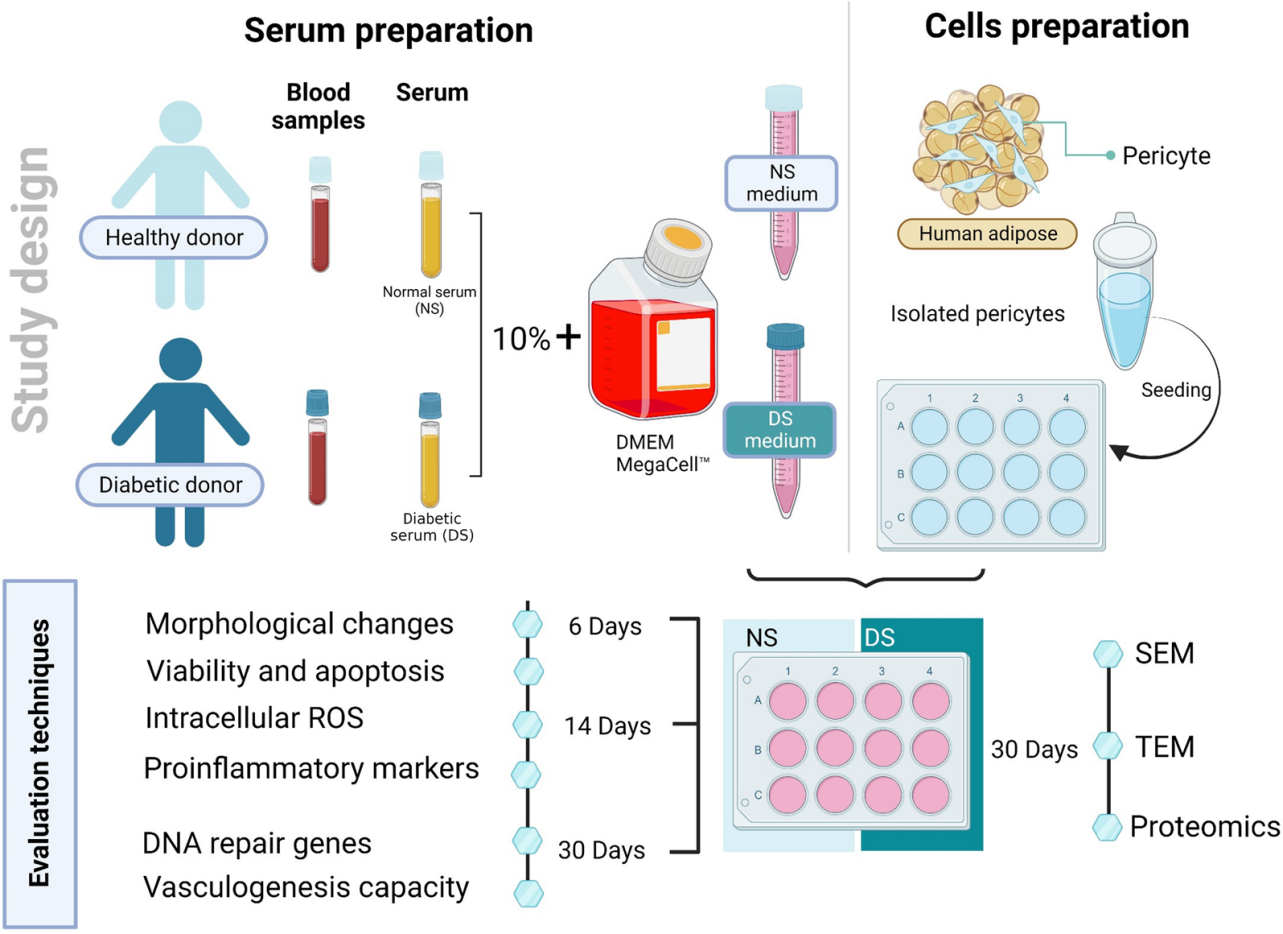
Schematic diagram of the study design showing the experimental details and evaluation methods. This figure was created using BioRender software

Primary PCs were isolated and characterized in our laboratory following a published protocol [17]. Briefly, PCs were thawed and cultured in their specific culture medium (Dulbecco’s Modified Eagle’s Medium (DMEM)-mega-cell supplemented with 10% FBS (Sigma-Aldrich, USA), 2 mM glutamine, 100 U/ml penicillin–streptomycin amphotericin, 1% non-essential amino acids (Lonza, Switzerland), 0.1 mM β-mercaptoethanol (Serva, Germany), and 5 ng/ml basic fibroblast growth factor (Gibco, USA). After reaching 70–80% confluency, PCs were washed and distributed to two groups. The first was cultured in PC culture medium in which 10% human pooled diabetic serum (DS) replacing conventional FBS. The second control group was cultured in PC culture medium supplemented with 10% human pooled normal serum (NS) instead of FBS. All experiments were performed after 6, 14, and 30 days of culture and in triplicate. Figure 1 provides a schematic diagram of the study design.

### Morphological analysis

Morphological examination of cells cultured in NS and DS at the designated timelines (days 6, 14, and 30) was done using Leica inverted microscope (Leica DMI8, Germany). The long-term effect of the diabetic microenvironment on the topography and ultrastructure of human adipose tissue-derived PCs was investigated using scanning and transmission electron microscopy (SEM and TEM) as previously described [17, 39]. Briefly, after culture in a medium supplemented with either 10% of NS or DS for 30 days, PCs were fixed with 0.1 M cacodylate-buffered 2% glutaraldehyde for 2 h at 4°C. The cells were washed with equal volumes of 0.4% sucrose and 0.2% cacodylate for 2 h before being fixed with equal volumes of 0.2% cacodylate and 2% osmic acid for 1 h. PCs were washed twice with distilled water, then dehydrated by incubating in an ascending series of ethyl alcohol for 5 min; followed by clearance with propylene oxide. Finally, the PCs were embedded in epoxy resin. The polymerized resin blocks were cut into semithin sections (1 μm thick) with an ultramicrotome (American Optical Co., USA). The resulting sections were then stained with methylene blue and azure mix and examined under a light microscope. Ultrathin sections (60 nm thick) using a diamond knife were mounted on perforated copper grids (Electron Microscopy Science, Hatfield, USA). Finally, the ultrathin sections were stained with uranyl acetate and lead citrate. Examinations and imaging were performed using TEM (TEM, Philips EM 208S, Tokyo, Japan) at an 80 kV acceleration voltage. For the study of the topography, PCs were processed using the same procedure as described for TEM till the step of dehydration with an ascending concentration of ethyl alcohol (30%, 50%, 70%, and 90%) for 5 min each. PCs were then rinsed with absolute ethyl alcohol three times for 3 min each and examined on the Formvar coating grids by SEM (Inspect S50; FEI, Holland).

### Apoptosis assay

Apoptosis assay was conducted on the PCs cultured in human NS and DS for 6, 14, and 30 days. Briefly, cells were stained with annexin and propidium iodide (PI) using annexin-V-FITC and PI apoptosis detection kit (Miltenyi Biotec Inc., USA) according to the manufacturer's instructions and as reported before [39–41]. Apoptosis markers were analyzed using FACS Calibur flow cytometer (Becton Dickinson, USA) and analyzed by CellQuest Pro software (Becton Dickinson, USA).

### Detection of ROS

The level of intracellular ROS of PCs was assessed after 6, 14, and 30 days of exposure to NS and DS. ROS measurement was conducted using DCFDA/H2DCFDA—Cellular ROS Assay Kit (Abcam, USA), following manufacturer’s instructions and as reported before [41–43].

### Investigating In vitro vasculogenesis using tube formation assay

To investigate the effect of the diabetic microenvironment on the angiogenic differentiation potential of human PCs, tube formation assay was performed using angiogenic starter kit (Gibco, USA) as previously described [44]. Briefly, PCs cultured in NS and DS for 6, 14, and 30 days were diluted in supplemented medium 200 and cultured on Geltrex® LDEV-free reduced growth factor basement membrane matrix-coated plates (Gibco, USA) at a density of 20,000 cells/cm^2^. Cells were incubated overnight at 37°C in a humidified atmosphere of 5% CO_2_. After 12 h of seeding, the cells were stained with 2 μg/ml of Calcein AM (Life Technologies, USA) and incubated at 37°C in a 5% CO_2_ atmosphere for 30 min. Finally, the cells were imaged at 5X magnification using phase contrast and fluorescence inverted microscopes (Leica DMI8, Germany). The total number of branches in three different fields of each well were counted and reported.

### Real-time qPCR

Total RNA was extracted from PCs cultured in NS and DS for 6, 14, and 30 days using PureLink® RNA Mini Kit (Life Technologies) according to the manufacturer's instructions. The total RNA was reverse transcribed into cDNA using RevertAid First-Strand cDNA synthesis kit (Thermo Scientific, USA), and quantitative Real-Time PCR assay was performed using SsoAdvanced™ Universal SYBR® Green Supermix (Bio-Rad) on the QuantStudio™ 12K Flex Real-Time PCR System (Applied Biosystems, Foster City, CA, USA). The relative gene expression of the pro-inflammatory markers ICAM-1, TNFα, IL-6, and TGF-β, and the DNA repair markers XRRCC5, TERF1, TERF2, CDKN1A and SIRT1, as well as vasculogenic genes VEGF-A and IGF-1 was normalized to β-actin gene and calculated by the comparative threshold (2^ΔΔCT^) method [44, 45].

### Proteomics analysis

#### Shotgun proteomics analysis

##### Preparation of cell protein lysate

Collected cells were quickly rinsed with PBS and centrifuged at 10,000 rpm. Cellular protein was obtained by replacing approximately 30 µl lysis solution (8 M urea, 500 mM Tris HCl, pH 8.5) with complete ultra proteases (Roche, Mannheim). Samples were incubated at 37°C for 1 h with occasional vortexing, then centrifuged at 12,000 rpm for 20 min. The lysate was assayed using BCA method (Pierce, Rockford IL) at Å562 nm prior to digestion.

###### Proteins tryptic digest

30 µg of cell protein lysate from each sample were subjected to solution digestion. The protein pellets were resuspended in 8 M urea lysis solution and reduced with 200 mM 1,4-dithiothreitol (DTT) for 30 min. Alkylation of cysteine residues was performed using 10 mM iodoacetamide for 30 min in dark area. Samples were diluted to a final concentration of 2 M urea in 100 mM Tris–HCl, pH 8.5 prior to digestion with trypsin. For endopeptidase digestion, modified porcine trypsin (Sigma, Germany) was added at a 30:1 (protein: protease mass ratio) and incubated overnight in a thermo-shaker at 600 rpm at 37°C [46]. Digested peptide solution was acidified using 90% formic acid to a final pH of 2.0. The resultant peptide mixture was cleaned up using stage tip as discussed earlier [47]. Peptides were assayed using BCA method (Pierce, Rockford IL) at Å562 nm prior to injection to be (1 µg /10 µl).

###### Nano-LC MS/MS analysis

Nano-LC MS/MS analysis was performed using TripleTOF 5600^+^ (AB Sciex, Canada) interfaced at the front end with Eksigent nanoLC 400 autosampler with Ekspert nanoLC 425 pump. Peptides were trapped on CHROMXP C18CL 5um (10 × 0.5 mm) (Sciex, Germany) in trap and elute mode. MS and MS/MS ranges were 400–1250 m/z and 170–1500 m/z, respectively. A design of a 120-min linear gradient 3–80% solution (80% ACN, 0.2% formic acid) was done. The 40 most intense ions were sequentially selected under data dependent acquisition (DDA) mode with a charge state of 2–5. For each cycle, survey full scan MS and MS/MS spectra were acquired at resolution of 35.000 and 15.000, respectively [48]. To ensure accuracy, external calibration was scheduled and run during sample batches to correct possible TOF deviation. Each sample was run into triplicates.

##### Proteomics data analysis

Mascot generic format (mgf) files were generated from raw file using script supplied by AB Sciex. MS/MS spectra were searched using X Tandem search algorithm within SearchGUI (Galaxy version 3.3.12) and Peptide shaker (Galaxy version 1.16.38) against Uniprot *Homosapiens* (Swiss-prot and TrEMBL database containing 226,953 proteins), with target and decoy sequences. The search of all fully and semitryptic peptide candidates were adjusted up to 2 missed cleavages with at least 6 amino acids. Precursor mass and fragment mass were identified with an initial mass tolerance of 20 ppm and 10 ppm, respectively. Carbamidomethylation of cysteine (+ 57.02146 amu) was considered as a static modification and oxidation at methionine (+ 15.995), acetylation of protein N-terminal and K (+ 42.01 amu), and pyrrolidone from carbamidomethylated C (-17.03 amu) as variable modification. To assure high-quality results, the false discovery rate (FDR) was kept at 1% at the protein level.

##### Statistical analysis

Before analysis, a probabilistic quotient normalization (PQN) was applied [49]. Proteins with NAs in two or more samples were removed from further analysis. A pair-wise approach was used to examine the signature of proteins in this study (i.e., PCs-DS6, PCs-DS14, and PCs-DS30 Vs. PCs-NS6, PCs-NS14, and PCs-NS30). We then performed a fold change (FC) analysis on the proteins found in all groups with two FC thresholds. Pathway enrichment analysis and gene ontology annotation with a *p*-value less than 0.05 and FDR 5% were applied to the significant proteins from the FC calculations and unique proteins for each group.

## Results

### The diabetic microenvironment altered the morphological and ultrastructural characteristics of adipose tissue PCs

**Fig. 2:**
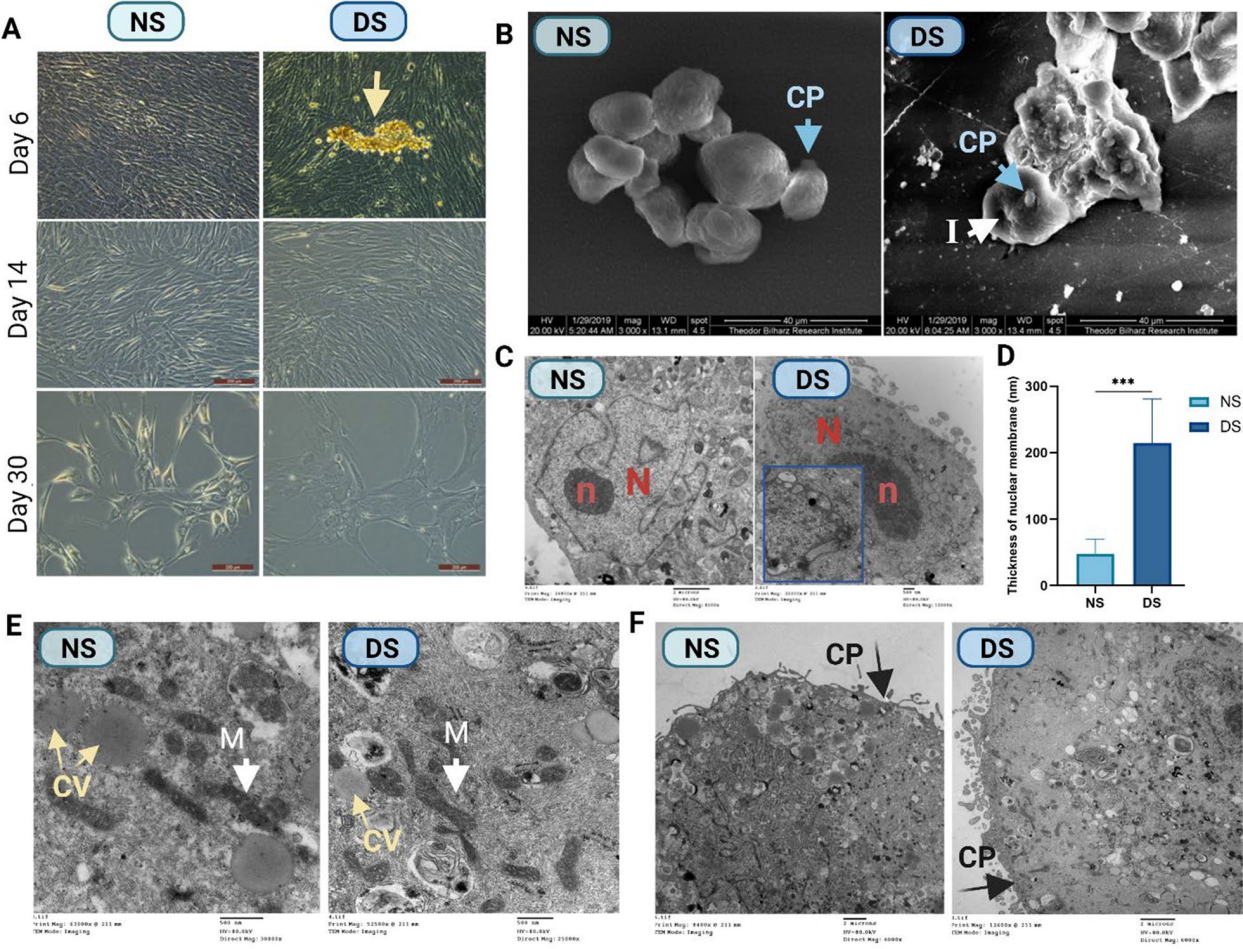
Microscopic images show the morphology of NS-PCs or DS-PCs. **A** Representative images of the morphological appearance of human PCs cultured in media supplemented with 10% NS or DS on days 6, 14, and 21. Yellow arrow indicates apoptotic cells aggregate, scale bar = 200 µm. **B** Scanning electron micrograph images for NS-PCs or DS-PCs at days 6, 14, and 30.), Scale bar = 40 µm, CP: cytoplasmic projections and I: invaginations. **C** transmission electron microscope images shows the ultrastructure of NS-PCs or DS-PCs at days 6, 14, and 30, N: nucleus and n: nucleolus. **D** Quantitative analysis of the nuclear membrane thickness, the data are presented as the mean ± standard deviation (***p < 0.0001), **E**, **F** transmission electron microscope images showed CV: cytoplasmic vesicles, M: mitochondria and CP

Under inverted microscopy, PCs cultured in NS appeared healthy with well-defined cell membranes and cell protrusions, while cells cultured in DS appeared flattened, with ill-defined edges, and a condensation of rounded yellowish bodies, which were most obvious at day 6 (Fig. 2A). On SEM examination, DS-PCs appeared condensed in cell aggregates, displayed cell membrane invaginations and deformed cytoplasmic projections (Fig. 2B). TEM analysis showed thicker nuclear membranes that characterized DS-treated PCs compared to NS-treated PCs (214.6 nm vs. 47.75 nm, respectively, *p* < 0.0001, 4.5-fold, Fig. 2C, D). DS-PCs showed pyknotic nucleus compared to the normal prominent eccentric nucleus with distinct nucleolus in NS-PCs (Fig. 2C). Mitochondria were more elongated in NS-PCs compared to DS-PCs, which had more circular mitochondria (Fig. 2E). Both NS-PCs and DS-PCs showed normal cytoplasmic vesicles (Fig. 2E). DS-PCs showed ruptured cytoplasmic projections from the cell boundary and detached microsomes compared to the normal projections in NS-PCs (Fig. 2F). DS-PCs showed detached microsomes.

### The diabetic microenvironment induces PCs apoptosis

**Fig. 3:**
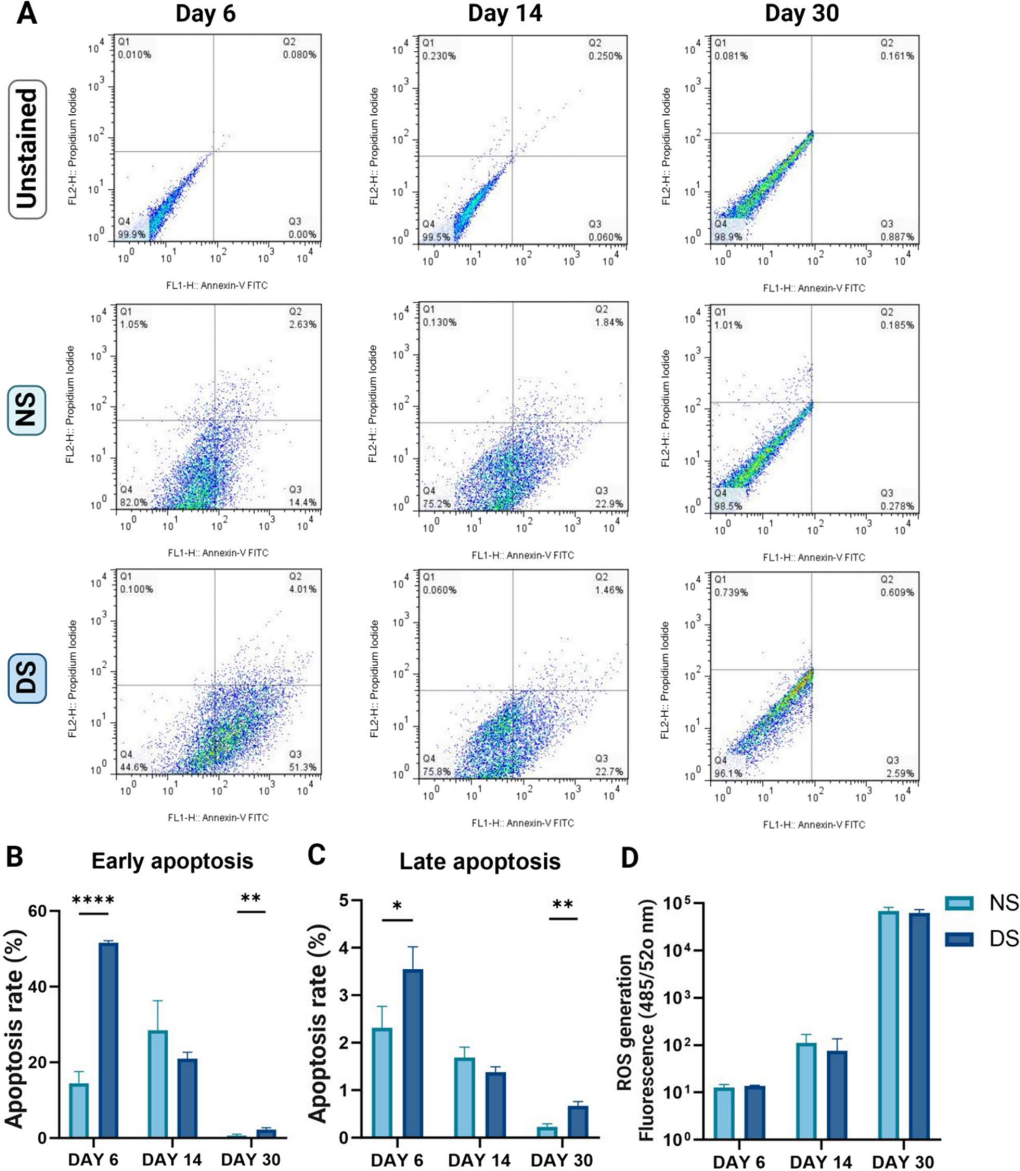
Assessment of cytotoxicity and the level of intracellular ROS for NS-PCs or DS-PCs at days 6, 14, and 30. **A** Flow cytometry analysis for apoptosis. The dot plot shows the following populations: necrotic (Q1-upper left), late apoptosis (Q2-upper right), early apoptosis (Q3-lower right), and viable cells (Q4-lower left). **B**, **C** Quantitative analysis of the apoptosis rate for NS-PCs or DS-PCs at days 6, 14, and 30. The data are presented as the mean ± standard deviation (*p < 0.05, ***p < 0.0001). **D** Intracellular ROS measurement assay for NS-PCs or DS-PCs at days 6, 14, and 30. The data are presented as the mean ± standard deviation

DS-PCs suffered a significantly higher apoptotic rate as shown by Annexin-PI assay compared to NS-PCs on day 6 (*p* < 0.0001, 3.6-fold) and day 30 (*p* < 0.01, 2.9-fold change), with no significant difference on day 14 (Fig. 3A–C).

### PCs treated with DS showed normal ROS levels

No significant difference in intracellular ROS production was observed between NS-treated and DS-treated PCs at the 3 measured time points, days 6, 14, and 30. Whereas ROS generation after 14 days in the NS-PCs group was significantly higher compared to that on day 6 (8.8-fold, *p* = 0.048). No significant difference was observed between day 6 and day14 in the DS treated group. However, on day 30, ROS levels were significantly higher compared to those observed on days 6 and 14 (4578.2-fold and 819.7-fold, respectively, *p* = 0.0019, Fig. 3D). Similarly, long-term incubation of PCs in NS showed significantly higher ROS level (day 30 compared to days 6 and 14, 5367.9-fold, *p* = 0.002 and 612.9-fold, *p* = 0.0187, respectively, Fig. 3B).

### The diabetic microenvironment abrogates the angiogenic potential of PCs

**Fig. 4:**
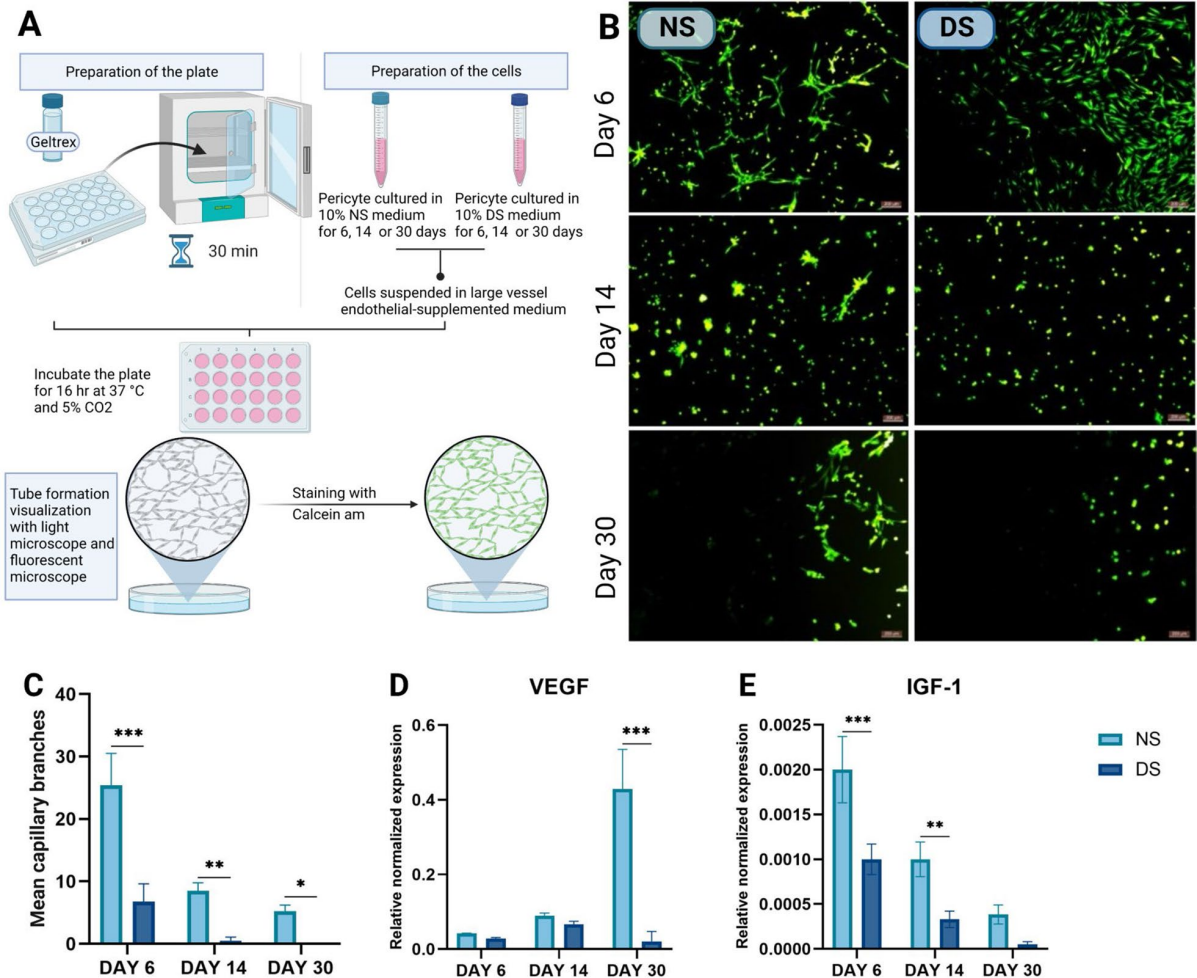
In vitro angiogenic tube formation assay. **A** Schematic diagram showing the tube formation assay. **B** Representative images showing in vitro tube formation for NS-PCs and DS-PCs at days 6, 14, and 30. Scale bar = 200 µm. **C** Quantitative analysis of the number of capillary-like branches on days 6, 14, and 30. **D** Quantitative real-time polymerase chain reaction analysis showing the expression levels of *VEGF* and (E) *IGF-1* mRNA. The data are presented as the mean ± standard deviation (*p < 0.05, **p < 0.01 ***p < 0.0001)

Tube formation assay (Fig. 4A) showed that NS-PCs displayed more robust tube formation on days 6, 14, and 30 compared to DS-PCs that showed short deformed tubes (Fig. 4B). Under both culture conditions, tube formation decreased over time from day 6 to day 30 (*p* < 0.0001, *p* < 0.01, and *p* < 0.05, respectively, Fig. 4C). Vascular endothelial growth factor A (*VEGF-A*) gene expression was significantly lower in DS-PCs compared to NS-PCs on day 30 (20-fold, *p* < 0.0001, Fig. 4D). Similarly, insulin growth factor 1 (*IGF-1*) gene expression in DS-PCs was lower than that of NS-PCs, and the reduction was especially pronounced on day 6 (twofold, *p* < 0.0001) and day 14 (threefold, *p* < 0.01, Fig. 4E).

### The diabetic microenvironment upregulates inflammatory marker expression

**Fig. 5:**
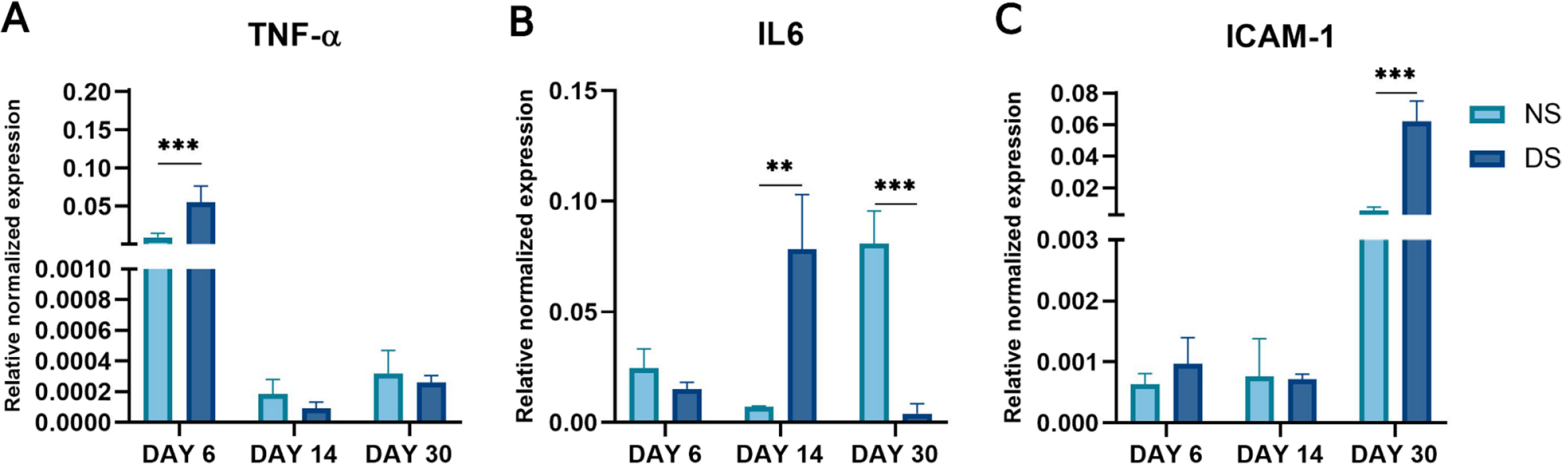
Quantitative real-time polymerase chain reaction analysis. The expression levels of mRNAs *TNFα* (**A**), *IL6* (**B**), and *ICAM* (**C**) in NS-PCs and DS-PCs at days 6, 14, and 30. The data are presented as the mean ± standard deviation (*p < 0.05, **p < 0.01 ***p < 0.0001)

DS-PCs showed significantly higher expression of inflammatory cytokines *TNF-α* (5.7-fold on day 6, *p* < 0.001, Fig. 5A), *IL-6* (~11-fold, *p* < 0.001, ~ 19-fold, *p* < 0.0001 on days 14 and 30, respectively, Fig. 5B), and *ICAM-1* ( ~ 10.7-fold, *p* < 0.0001 on day 30, Fig. 5C) compared to NS-PCs.

### Dysregulation of DNA repair genes in PCs cultured in DS

**Fig. 6:**
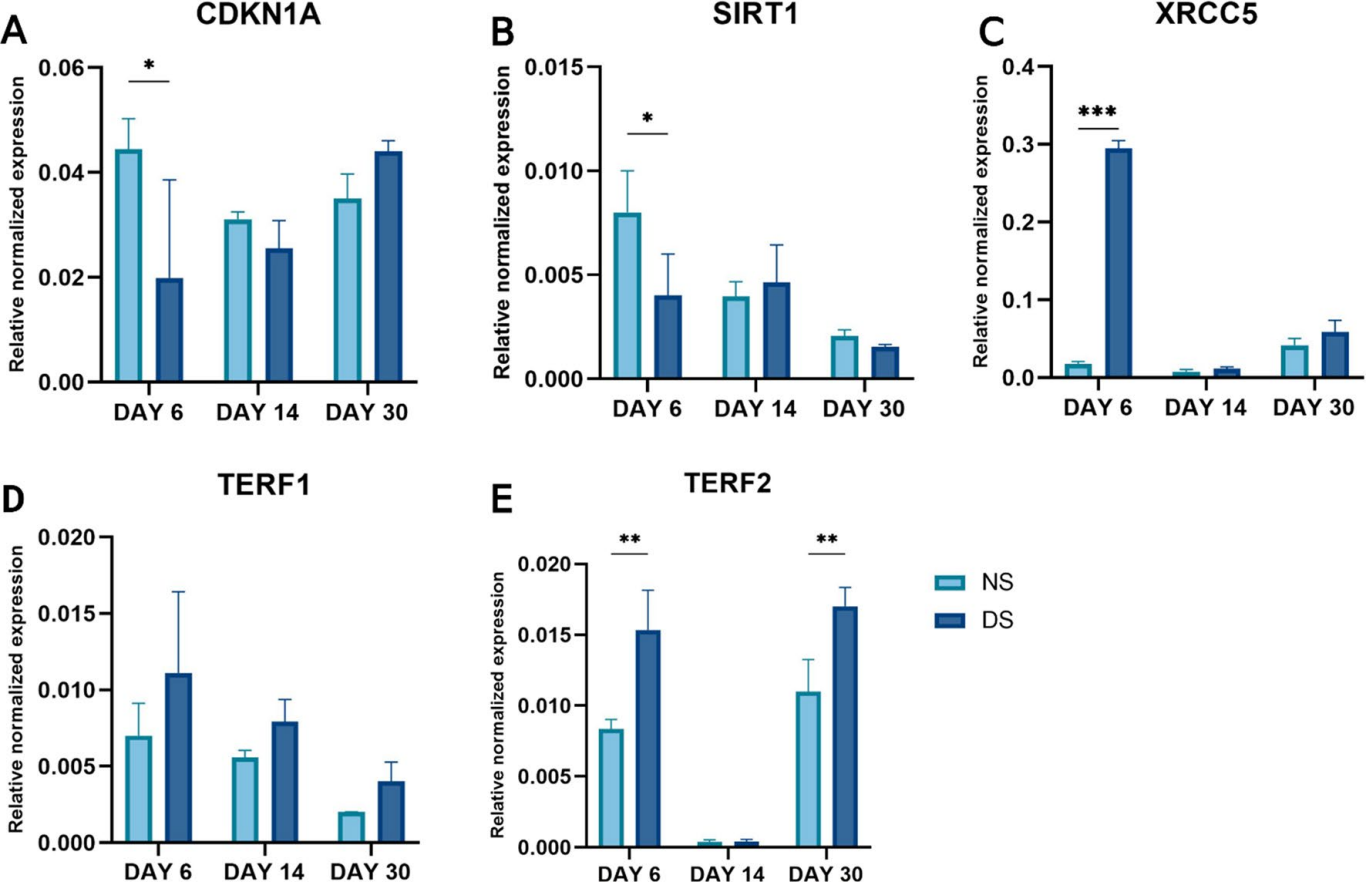
Quantitative real-time polymerase chain reaction analysis. The expression levels of mRNAs *CDKN1A* (**A**), *SIRT1* (**B**), *XRCC5* (**C**), *TERF1* (**D**), and *TERF2* (**E**) in NS-PCs and DS-PCs at days 6, 14, and 30. The data are presented as the means ± standard deviation (*p < 0.05, **p < 0.01 ***p < 0.0001)

Quantitative analysis of DNA repair genes showed significant downregulation in *CDKN1A* (*p* < 0.05) and *SIRT1* (twofold, *p* = 0.04) expression on day 6 (Fig. 6A, B) in DS-PCs with no significant difference on days 14 and 30. On the other hand, *XRCC5* showed significantly higher expression in DS-PCs in comparison to NS-PCs on day 6 (16 fold, *p* < 0.0001, Fig. 6C) with no significant difference on days 14 and 30. However, there was no significant difference in the expression of *TERF1* between NS- and DS-PCs on days 6, 14, and 30 (Fig. 6D). Additionally, *TERF2* was significantly increased in DS-PCs on days 6 and 30 (1.8 fold, *p* < 0.01, 1.5-fold, *p* < 0.01, respectively, Fig. 6E).

### Proteomic analysis

**Fig. 7:**
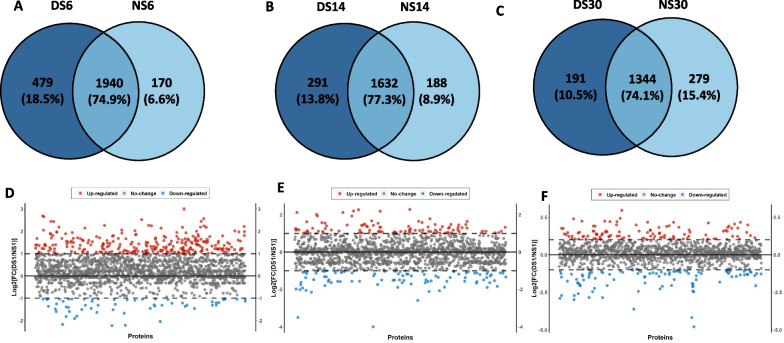
Proteomic analysis of NS-PCs and DS-PCs at days 6, 14, and 30

**Fig. 8:**
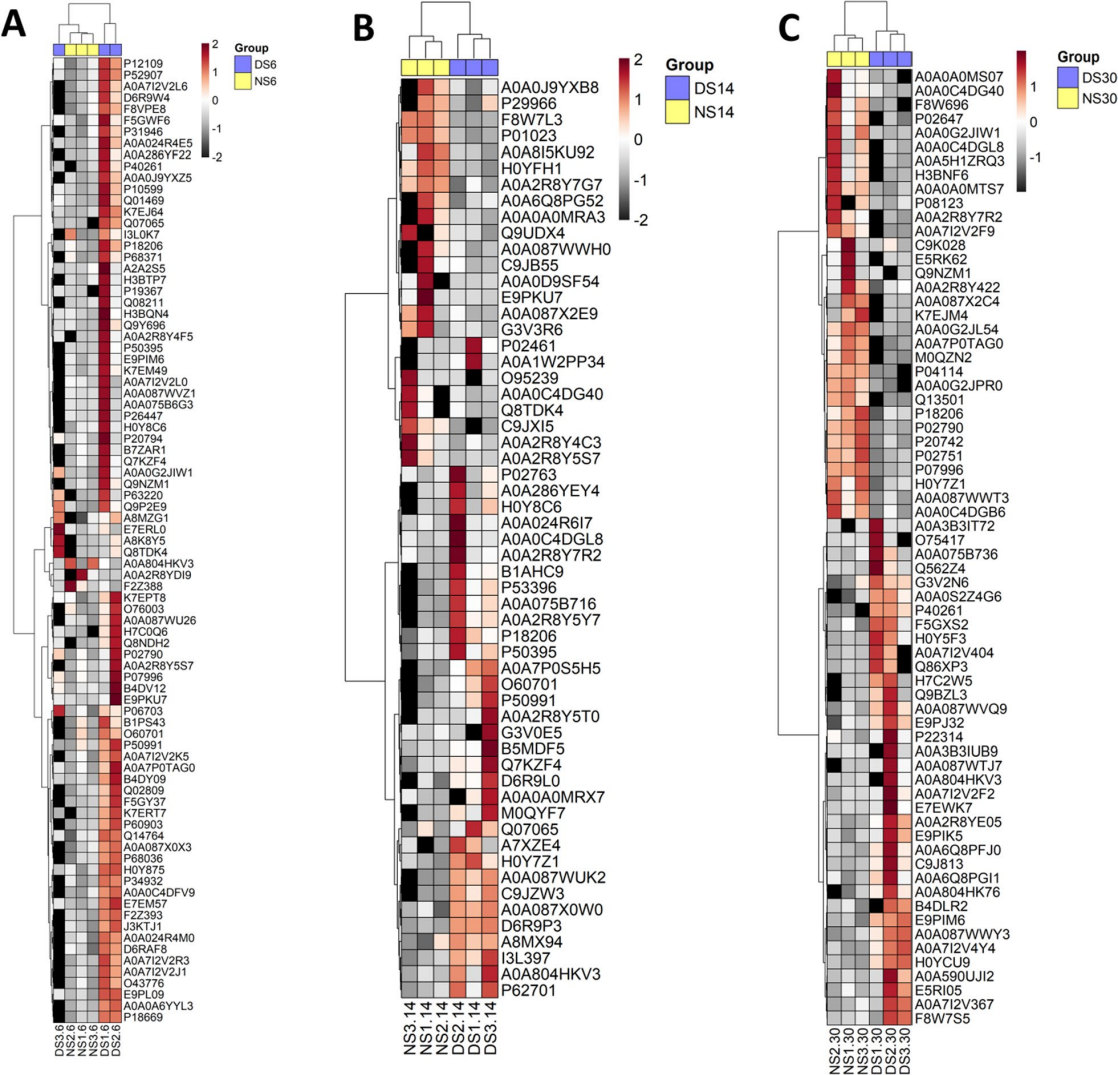
Heat maps of NS-PCs and DS-PCs at days 6, 14, and 30

Proteomics analysis showed unique 479 (18.5%) proteins in DS-PCs at day 6, 1940 (74.9%) shared proteins with NS-PCs, and 170 (6.6%) unique proteins for NS-PCs at day 6 (Fig. 7A). After 14 days of culture, DS-PCs showed unique 291 (13.8%) proteins, 1632 shared proteins (77.3%) with NS-PCs, and unique 188 proteins (8.9%) for NS-PCs at day 14 (Fig. 7B). PCs exposed to diabetic conditions for 30 days showed unique 191 (10.5%) proteins, and shared 1344 (74.1%) proteins with NS-PCs, and unique 279 (15.4%) proteins for PCs exposed to normal serum for 30 days (Fig. 7C). Scatter plot showing differences in protein abundances between NS-PCs and DS-PCs at days 6,14, and 30 (Fig. 7D–F). Heat maps for proteomics analysis for the three time lines respectively are shown in (Fig. 8A–C). PCs showed unique 247 (41%), 119 (19.7%), and 145 (24%) proteins after 6, 14, and 30 days of exposure to diabetic serum. Additional file 1: Fig. 1A shows 37 shared (6.1%) proteins between days 6 and 14, 23 (3.8%) shared proteins between days 6 and 30, 21 (3.5%) between days 14 and 30 and 11 (1.8%) proteins between days 6, 14, and 30. Heat map for the significant proteins at the three timelines was performed as well (Additional file 1: Fig. 1B–D). Classification of expressed proteins in both NS-PCs and DS-PCs at 6, 14, and 30 days is provided in (Additional file 2: Table 1).

## Discussion

We have previously reported the morphological, topographical, and ultrastructural characteristics of PCs obtained from adipose tissue under normal physiological conditions [7, 17, 50]. Herein, we report that PCs exhibit significant structural changes that could impact their physiological functions, when exposed to the diabetic microenvironment in patients’ serum. Cytoplasmic projections are a defining feature of PCs [51]. The deformed projections and topographic invaginations observed in the DS-PCs were reported to be associated with down regulation of expression of cell surface binding proteins, which are responsible for maintaining cell rigidity [52]. On the other hand, high expression of different forms of tubulins, including tubulin beta 4B chain and tubulin beta chain after 6 and 30 days respectively in DS-PCs may present resistance mechanisms for PCs to maintain the integrity of their cellular structure [53, 54].

The early stages of diabetic complications, such as retinopathy, are characterized by an increase in the thickness of the basement membrane and loss of PCs. Focal adhesions, which are complex protein structures, play a crucial role in the assembly and disassembly of the cytoskeleton, regulating cell polarity and migration [55, 56]. Our study reports the loss of PCs when exposed to diabetic conditions, and is associated with upregulation of the cell migration and motility proteins thrombospondin-1 (TSP1) and transforming growth factor beta (TGF-β) and downregulation of various focal adhesion proteins protocadherin 7 (PCDH7) and fibronectin. TSP1 is known to impact angiogenesis, cell adhesion, proliferation, migration, and cell survival [57]. It was reported to induce the disassembly of focal adhesions in bovine aortic ECs [58, 59], which could potentially affect the PCs' ability to adhere to the endothelium. Additionally, TSP1 has been shown to block nitric oxide (NO)-stimulated vascular smooth muscle cell relaxation [60].

In our study, TSP1 was upregulated after 6 days of exposure to DS. This upregulation may be associated with poor adhesion potential of PCs, and was accompanied by the upregulation of protein inhibitor of neuronal nitric oxide synthase (PIN). TSP1 upregulation along with TGF-β upregulation after 6 days of exposure to diabetic microenvironment may be associated with weak adhesion of PCs to the endothelium. TGF-β was also reported to have a critical role in the pathogenesis of T2DM [61] and was found to be involved in focal adhesion protein regulation as protein phosphatase 1 and paxillin, resulting in increased cell motility and migration [62].

Collectively, our data show upregulation of focal adhesion and cytoskeleton and ECM organizing proteins at the early exposure to diabetic microenvironment with significant downregulation of these proteins after long term exposure indicating possible primary protective response, but eventually loss of this protective potential and increased risk of PCs loss from the endothelium. Vinculin, GTPase-activating-like protein (IQGAP1) and peptidyl-prolyl cis–trans isomerase (PPIase) were all upregulated after 6 and 14 days. However, after 30 days of prolonged exposure to diabetic microenvironment, they were all downregulated. Fibronectin was also upregulated at day 14 and downregulated at day 30, while the level of PCDH7 was compromised at day 30. Vinculin is a cytoskeletal protein important in cell-to-cell and cell-to-ECM interactions [63, 64]. GTPase-activating-like protein (IQGAP1) plays a role in the organization of the actin cytoskeleton and cellular adhesion [65]. Studies have shown that its expression is decreased in podocytes in human diabetic nephropathy [65, 66]. Peptidyl-prolyl cis–trans isomerase (PPIase) plays an essential role in regulating both the dynamic cell-ECM interaction and the mechanical properties of different ECM components [67, 68]. Fibronectin, an endothelium-derived glycoprotein plays a critical role in ECM formation and cell motility [69, 70] and is an important PC's adhesion protein [71]. Its disruption may contribute to poor adhesion of PCs and increased risk of loss and vascular complications in diabetic patients [70, 71]. PCDH7 on the other hand is an adhesion protein involved in cell–cell adhesion [72]. Our data thus suggest that compromised adhesion proteins play a critical role in the pathogenesis of T2DM by compromising the interaction between PCs and ECs, eventually resulting in PCs loss. T2DM is characterized by apoptosis-dependent loss of β-cell mass, which leads to a reduction in pancreatic insulin secretion. The diabetic microenvironment in our study promoted PC apoptosis. DS-PCs displayed lower gene expression of CDKN1A in DS-PCs at day 6 [73], and upregulation of TNF-α along with an increase in TERF2 expression. This is in accordance with previous studies which reported that TNF-α can trigger apoptosis through TERF2 and activation of NF-kB transcription factors [74, 75]. Additionally, it has been demonstrated that the blocking of NF-kB activation using anti-ROS antibodies not only inhibited PC apoptosis, but also improved vascular dysfunction [76]. Our data indicate that XRCC5 gene expression is linked to increased TERF2 gene expression, which is in line with previous findings reported by Hu L et al. [65]. The finding that chloride intracellular channel protein-4 (Clic4) in DS-PCs was upregulated at day 6 may contribute to the high apoptotic rate, as Clic4 has been reported to reduce BCL-2 and sensitizes β-cells to apoptosis. High expression of both serine/threonine protein phosphatases at days 6 and 30 and the apoptosis facilitator Bcl-2-like protein 14 at day 30 may be attributed to a higher rate of apoptosis in DS-PCs. However, DS-PCs unexpectedly showed downregulation of serine/threonine protein phosphatase at day 14. Additionally, another apoptosis signaling pathway, caspase-7, showed a 2.4-fold higher expression in DS-PCs compared to NS-PCs at day 30.

Angiogenesis and maintaining the blood vessel integrity is the most essential physiological role of PCs [17, 77], where angiogenesis disruption was reported in diabetic patients [3, 8]. This is likely due to the high prevalence of anti-pericyte autoantibodies in type 2 diabetic patients, which are considered a main cause of blood vessel damage [78]. Our data show that the diabetic microenvironment impedes the angiogenic potential of PCs in a time dependent manner, as indicated by a decrease in the expression of the VEGF-A gene and deformed capillary-like structures. The upregulation of the TSP, platelet factor 4 (PF4) and actin-related protein 2/3 complex proteins in DS-PCs at day 6 and the downregulation of collagen type XXI alpha 1 chain (COL21A1) protein in DS-PCs at day 14 are also associated with impaired angiogenic potential. TSP1 was reported to have an inhibitory effect on angiogenesis [79, 80]. PF4 could be associated with vascular disturbance in the early stages of diabetes, but this effect disappears after longer exposure to the diabetic microenvironment. This is in line with a previous study that reported that PF4 has been found in the blood vessel wall after minutes of endothelium's injury in rabbits [81]. The upregulation of actin-related protein 2/3 complex in DS-PCs at day 6 may be associated with impaired actin polymerization, known to contribute to neural and angiogenic alterations observed in diabetic patients [82, 83]. The downregulation of COL21A1 in DS-PCs at day 14 may indicate disruption of the vascular network as previously reported [84].

We have previously reported that human adipose tissue-derived PCs exhibited cytoplasmic vesicles known as caveolae, important for maintaining the functionality of PCs [17]. We found caveolin-1, an important protein component of caveolae [17], to be upregulated in DS-PCs at day 14, leading to the proposal that exposure of the PCs to the diabetic microenvironment may present an early defense mechanism to secure their angiogenic functionality. Caveolin-1 was reported to contribute to the first steps of altered shear stress conditions in blood vessels and was also reported to regulate eNOS function [85, 86]. This may explain the upregulation of NO synthase in DS-PCs in a short time window at day 14 in our study. This upregulation was not reported earlier at days 6 or later at day 30, and NO synthase disappeared on days 6 and 30.

Inflammation was reported to play a central role in the pathophysiology of T2DM [87]. The inflammatory profile of DS-PCs showed that *TNF-α* stimulates the production of *IL-6* cytokines as well as *ICAM-1* molecules through activation of NFκB [88]. It is also evident that this effect occurs in a time-dependent manner as the increase of *TNF-α* on day 6 followed by an *IL-6* increase on day 14 and *ICAM-1* on day 30. On the other hand, *SIRT1* was reported to have a protective potential on β-cells through the suppression of NFκB [89]. Herein, the notable lower expression of *SIRT1* in DS-PCs confirms the activation of NFκB upon exposure to diabetic conditions. On the other hand, the decreased inflammatory response via the macrophage migration inhibitory factor (MIF) inhibition in DS-PCs could indicate a resistance potential of these cells [90]. MIF is a T cell-derived cytokine that inhibits macrophage migration causing their accumulation in different hypersensitivity conditions [90–92].

Although human serum is a physiologically relevant representative of the diabetic microenvironment, pooled serum samples in this study were collected from patients treated with insulin with or without metformin, which may impact the analysis. Insulin was reported to have a crucial role in maintaining the functionality of and integrity of vascular system and mediating the function of the vascular smooth muscle cells, ECs and PCs [93]. Insulin receptor knockdown showed decreased sprouting of brain PCs [94]. Metformin was also reported to prevent brain PCs apoptosis and relieve tight junction loss [95]. As human serum is complex and rich in many proteins, growth factors and enzymes, distinguishing the effects of insulin or metformin treatment, or any other confounding factors can be a challenge. However, our data on various deleterious effects of the diabetic serum on PC structure and function, such as apoptosis and compromised angiogenesis, are in line with the deleterious effects of diabetes itself, and not its medications.

Cross-linking between ribosomal proteins (RPs) or ribosomal biogenesis and intracellular ROS level has been previously reported that [96, 97]. RPs are essential for cellular redox homeostasis. In this study, DS-PCs showed upregulation of 40S RPS9, 60S RPL32, 60S RPL9, 40S RPS3, 40S RPS2, 60S RPP0, 60S RPL6, 60S acidic RPP2, 60S RPL4, 40S RPS11, 40S RPS21, and 60S RPL14 at day 6. DS-PCs showed high expression of other RPs, including 40S RPS19, 40S RPS17, 60S RPL9 RPL19, 40S RPS20, 40S RPS2, 40S RPS3, 60S RPL4, and 40S RPS4 at day 14, and high expression of 60S acidic RPP0, 60S RPL4, 60S RPL13a, 60S RPL10a at day 30. These data allude to a resistance mechanism, by which PCs are protected from the toxic diabetic microenvironment, in an attempt to maintain normal physiological levels of intracellular ROS level while exposed to diabetic conditions.

Along with upregulation in alcohol dehydrogenase class 3 (ADH3), these data suggest a protective mechanism to resist the toxic diabetic microenvironment, perhaps via formaldehyde detoxification induction. ADH3 has a critical role in formaldehyde detoxification in the human body thanks to its glutathione-dependent formaldehyde dehydrogenase activity [98, 99]. The involvement of formaldehyde in different metabolic age-related diseases such as dementia, glaucoma, declined memory, stroke, and T2DM has been previously reported [100–105].

40S ribosomal protein S6 (RPS6) is one of the ribosomal proteins that have essential roles in different cellular processes [106]. rpS6-deficient mice have shown a decrease in both pancreatic and circulating insulin, as well as a prolonged response to hyperglycemic conditions [107]. Additionally, a low protein diet during pregnancy in rats has been shown to produce unhealthy fetuses with decreased pancreatic cell proliferation, insulin content, and smaller islets [108]. DS-PCs showed low expression of RPS6. On the other hand, RPS6 was reported to cause metabolic abnormalities in type 2 diabetic patients and may also play a role in reducing focal adhesion through the PI3K-Akt-mTOR signaling pathway.

14–3-3 proteins (14-3-3 protein theta and 14-3-3 protein beta/ alpha) were found to bind to insulin receptor substrate-1 (IRS-1) and play a role in insulin desensitization [109]. The upregulation of 14-3-3 proteins and prolyl endopeptidase fibroblast activating protein (FAP) in DS-PCs in our study purports a role in diabetic complications. Prolyl endopeptidase FAP was shown to have a proteolytic impact on fibroblast growth factor 21 (FGF21) decreasing its lifetime [110–113]. FGF21 is a class of endocrine hepatokines secreted from the liver and adipose tissue [114, 115] and has a crucial role in protecting against insulin resistance. FAP inhibition has been proposed as a novel target to maintain the activity of FGF21 in some animal models [112, 116–118].

## Conclusion and clinical implications

In summary, the current study aimed to investigate the impact of a diabetic microenvironment on PCs, which are important cells for maintaining the integrity and functionality of blood vessels. Data showed that DS impaired the morphology and ultrastructure of PCs and compromised their binding into the endothelium which increase the risk of PC's loss and associated diabetic vascular complications. Exposure to diabetic conditions increased in apoptotic rate in PCs and decreased their angiogenic potential. On the other hand, PCs exhibited resistance mechanisms upon exposure to diabetic conditions as shown by maintained intracellular ROS level, high expression of some RPs and upregulation of focal adhesion proteins at early exposure. Our data show that exposure to the diabetic microenvironment compromises the structure and function of adipose tissue-derived PCs. However, upon exposure to diabetic microenvironment, PCs showed a resistance mechanism that probably aims at protecting the cells from oxidative damage by maintaining the level of intracellular ROS. Another resistance mechanisms include upregulation of RPs, focal adhesion proteins and ECM organizing proteins is to maintain cellular integrity in the stressful diabetic microenvironment. This profile may also serve as biomarkers to predict subclinical diabetes, and provides a window to therapeutically target for EC’s dysfunction, and associated diabetes complications. While this work focuses on the deleterious effect of the diabetic microenvironment on the regenerative capacities of PCs, obtaining clinically relevant data would benefit from evaluating this effect in a three dimensional system. Inclusion of other relevant cellular components, such as ECs and macrophages would prefer a more physiologically relevant model. Additional molecular investigations are needed to uncover the therapeutic targets for vascular complications associated with PCs loss. Analysis of genetic and epigenetic alterations in complex organoids in DS would provide valuable insight into the pathophysiology and progress of diabetic complications, and provide early intervention strategies to achieve better prognosis of T2DM.

### Supplementary Information

**Additional file 1**. Supplementary Figure 1A–D.

**Additional file 2**. Supplementary Table 1.

## Data Availability

All data presented in this paper are available and present in the text. MS proteomics data were deposited to the ProteomeXchange Consortium through the PRIDE repository with the dataset identifier PXD048187.

## Abbreviations

PCs: Pericytes
ECs: Endothelial cells
T2DM: Type 2 diabetes mellitus
DS: Diabetic serum
DS-PCs: PCs cultured in DS
NS-PCs: PCs cultured in normal serum
*VEGF-A*: Vascular endothelial growth factor
*IGF-1*: Insulin-like growth factor 1
TSP1: Thrombospondin-1
PF4: Platelet factor 4
COL21A1: Collagen type XXI alpha 1 chain
Clic4: Chloride intracellular channel protein-4
BCl-2: B-cell lymphoma 2
*CDKN1A*: Cyclin-dependent kinase inhibitor 1A
*SIRT1*: Sirtuin
*XRCC5*: X-ray repair cross-complementing protein 5
*TERF2*: Telomeric repeat-binding factor 2
*ICAM1*: Intercellular adhesion molecule
*IL-6*: Interleukin-6
*TNF-α*: Tumor necrosis factor alpha
TGF-β: Transforming growth factor-β
PCDH7: Protocadherin 7
ROS: Reactive oxygen species
ECM: Extracellular matrix
IQGAP1: Isoleucine-glutamine Ras GTPase-activating-like protein
DM: Diabetes mellitus
GULT-1: Glucose transporter 1
FBS: Fetal bovine serum
NS: Normal serum
PI: Propidium iodide
FITC: Fluorescein isothiocyanate
FACS: Fluorescent activated cell sorter
SEM: Scanning electron microscopy
TEM: Transmission electron microscopy
DCFDA: Dichlorodihydrofluorescein diacetate
qPCR: Quantitative polymerase chain reaction
RNA: Ribonucleic acid
cDNA: Complementary deoxyribonucleic acid
TERF1: Telomeric repeat binding factor 1
PBS: Phosphate-buffered saline
Rpm: Revolutions per minute
HCl: Hydrochloric acid
BCA: Bicinchoninic acid
DTT: 1,4-Dithiothreitol
Tris: Trisaminomethane
Nano-LC MS/MS: Nanoscale liquid chromatography coupled to tandem mass spectrometry (nano LC–MS/MS)
LC: Liquid chromatography
DDA: Data-dependent acquisition
TOF: Time of flight
mgf: Mascot generic format
GUI: Graphical user interface
TrEMBL: Translated European Molecular Biology Laboratory
Ppm: Parts per million
PQN: Probabilistic quotient normalization
NAs: Nicotianamine
FC: Fold change
FDR: False discovery rate
NO: Nitric oxide
GTP: Guanosine triphosphate
PPIase: Peptidyl-prolyl cis–trans isomerase
NFκB: Nuclear factor kappa-light-chain-enhancer
MIF: Migration inhibitory factor
RPs: Ribosomal proteins
ADH3: Alcohol dehydrogenase class 3
RPS6: 40S ribosomal protein S6
PI3K-Akt-mTOR: Phosphatidylinositol 3-kinase (PI3K)/protein kinase B (AKT)/mammalian target of rapamycin
IRS-1: Insulin receptor substrate-1
FAP: Fibroblast activating protein
FGF21: Fibroblast growth factor 21
