# Supplementary figures and images for: The cross talk between type II diabetic microenvironment and the regenerative capacities of human adipose tissue-derived pericytes: a promising cell therapy
Source: Stem Cell Res Ther. 2024 Feb 8;15:36. doi: 10.1186/s13287-024-03643-1 (PMC10854071; doi:10.1186/s13287-024-03643-1)

## Slide 1
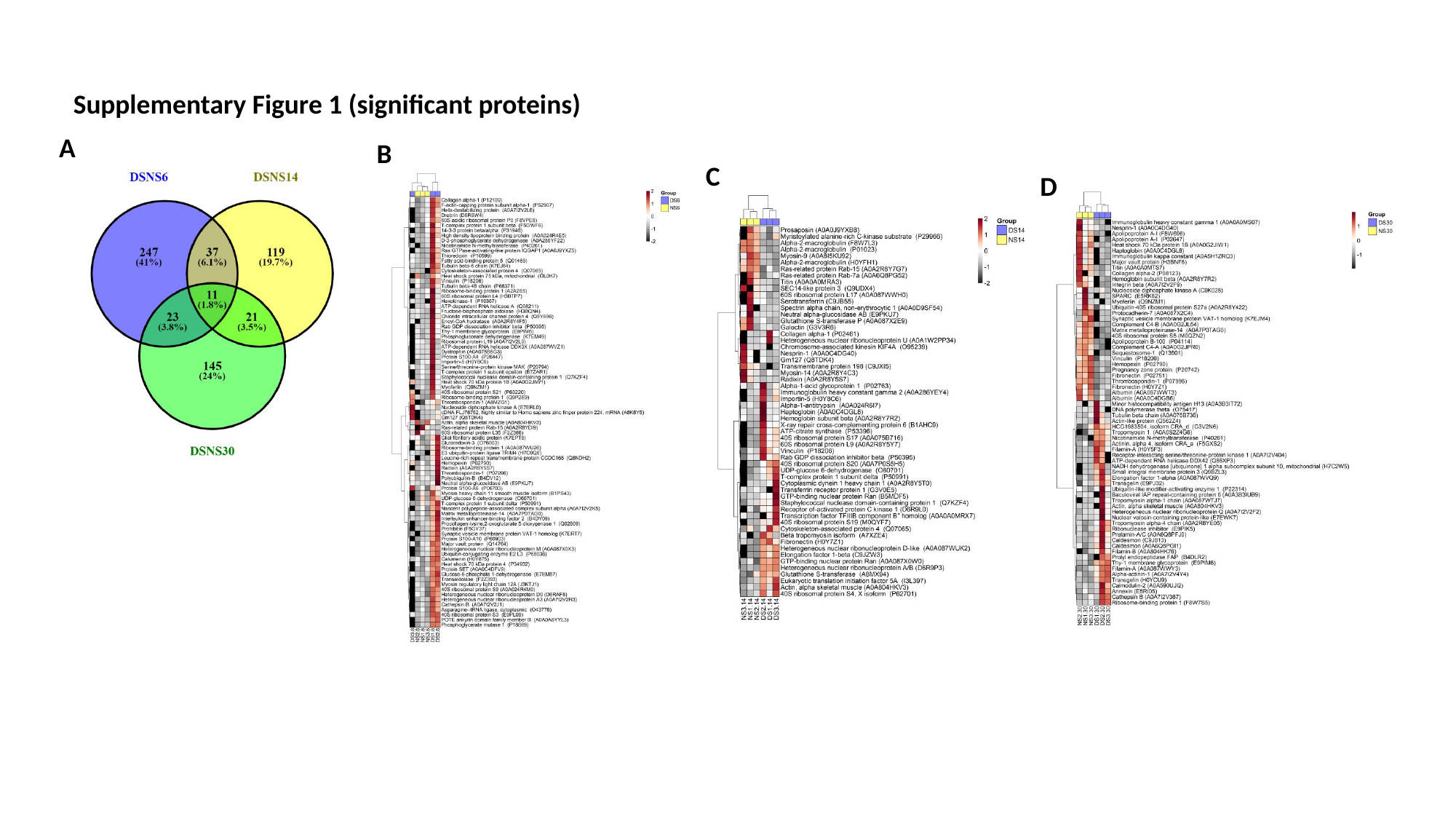

Supplementary Figure 1 (significant proteins)
A
B
C
D

Supplement: Supplementary file 1 — Additional file 1. Supplementary Figure 1A–D. [file 13287_2024_3643_MOESM1_ESM.pptx]
