# Supplementary material for: The cross talk between type II diabetic microenvironment and the regenerative capacities of human adipose tissue-derived pericytes: a promising cell therapy
Source: Stem Cell Res Ther. 2024 Feb 8;15:36. doi: 10.1186/s13287-024-03643-1 (PMC10854071; doi:10.1186/s13287-024-03643-1)
Supplement: Supplementary file 2 — Additional file 2. Supplementary Table 1. [file 13287_2024_3643_MOESM2_ESM.docx]

Supplementary Table 1: Classification of expressed proteins in both NS-PCs and DS-PCs at 6, 14 and 30 days.

| **Protein** | **Involved pathway/s** | **Result (up or down regulation) Log2.FC.** | | |
| --- | --- | --- | --- | --- |
|  |  | **Day 6** | **Day 14** | **Day 30** |
| Serine/threonine-protein phosphatase | Apoptosis  Cellular senescence  mTOR signaling pathway | Up/ 2.66 | Down/ -1.93 | Up/ 1.23 |
| Apoptosis facilitator Bcl-2-like protein 14 | Apoptosis | − | − | Up/ 1.58 |
| Caspase-7 | Intrinsic Pathway for Apoptosis | − | Down/ -1.16 | Up/ 2.4 |
| Cathepsin B | Apoptosis  NOD-like receptor signaling pathway | Up/ 1.6 |  | Up/ 1.2 |
| Desmoglein-2 (Cadherin family member 5) (HDGC) | Apoptotic cleavage of cell adhesion Proteins | − | Down/ -1.0 | − |
| Thrombospondin-1 | Focal adhesion  ECM-receptor interaction  Rap1 signaling pathway | Up/ 1.03 | − | Down/ -1.7 |
| Talin-2 | Rap1 signaling pathway | Down/ -1.14 | − | − |
| Transforming growth factor-beta | Focal adhesion | Up/ 1.0 | − | − |
| Na+/K+-ATPase | cGMP-PKG signaling pathway | Up/ 2.45 | − | − |
| Vinculin (Metavinculin) (MV) | Adherens junction  MAPK1/MAPK3 signaling  Smooth Muscle Contraction | Up/ 1.39 | Up/ 1.33 | Down/ -1.26 |
| Prolyl endopeptidase FAP |  | Up/ 1.07 | Up/ 1.39 | Up/ 1.25 |
| Synaptic vesicle membrane protein VAT-1 homolog | Immune System  Innate immune system  Neutrophil degranulation | Up/ 1.29 | Down/ -1.20 | Down/ -1.05 |
| Alcohol dehydrogenase class 3 (ADH3) | Metabolic pathways  Drug metabolism - cytochrome P450 | Up/ 1.14 | Up/ 1.06 | Up/ 1.07 |
| Src substrate cortactin (Amplaxin) | Tight junction | Up/ 1.5 | − | − |
| Protein phosphatase 2 | Tight junction  PI3K-Akt signaling pathway  Hippo signaling pathway | Up/ 1.7 | − | − |
| Cell division control protein 42 homolog | Regulation of actin cytoskeleton  Tight junction  Rap1 signaling pathway  p38 MAPK signaling pathway | Up/ 1.9 | − | − |
| Guanine nucleotide-binding protein subunit beta-4 | PI3K-Akt signaling pathway | Up/ 1.4 | − | − |
| Phosphatidylinositol-3,5-bisphosphate 3-phosphatase (Myotubularin-related protein 8) | Metabolic pathways | Up/ 1.2 | − | − |
| Protein phosphatase 1 regulatory subunit 12A | Regulation of actin cytoskeleton  Focal adhesion  Vascular smooth muscle contraction | − | Down/ -1.0 | − |
| Actin-related protein 2/3 complex subunit 4 | Regulation of actin cytoskeleton  Tight junction | Up/ 1.4 | − | − |
| 40S ribosomal protein S6 | PI3K-Akt signalling pathway  PI3K-Akt-mTOR-signaling pathway  Focal adhesion | Down/ -1.6 | − | − |
| 60S ribosomal protein L17 | Cellular responses to stress  Cellular responses to stimuli | − | Down/ -1.75 | − |
| Membrane-associated guanylate kinase inverted 3 | Rap1 signaling pathway | − | Down/ -1.66 | Up/ 1.91 |
| protein inhibitor of neuronal nitric oxide synthase (PIN) | Metabolism of proteins  Cellular responses to stress  Signaling by Rho GTPases | Up/ 1.33 | − | − |
| Fibronectin | Focal adhesion  ECM-receptor interaction  AGE-RAGE signaling pathway in diabetic complications | − | Up/ 1.17 | Down/ -1.28 |
| GTPase-activating-like protein (IQGAP1) | Adherens junction Regulation of actin cytoskeleton | Up/ 2.19 | Down/ -1.63 | − |
| Protocadherin-7 | Focal adhesion | − | − | Down/ -1.3 |
| Integrin beta | Focal adhesion | − | − | Down/ -2.1 |
| Platelet factor 4 | Common Pathway of Fibrin Clot Formation  Platelet activation, signaling and aggregation  Clotting Cascade | Up/ 1.95 | − | − |
| Caveolin-1 | Focal adhesion  RHO GTPase cycle | − | Up/ 1.47 | − |
| Nitric oxide synthase | Pathways of neurodegeneration - multiple diseases  Arginine and proline metabolism | − | Up/ 1.18 | − |
| G-protein coupled receptor |  |  |  | Down/ -1.09 |
| Collagen alpha-1(VI) chain | PI3K-Akt signaling pathway  Focal adhesion | Up/ 1.15 | − | − |
| Collagen alpha-2(V) chain | Protein digestion and absorption  Extracellular matrix organization  Collagen formation | Up/ 1.22 | − | − |
| Collagen alpha-3(VI) chain | ECM-receptor interaction  Focal adhesion | Down/ -2.22 | − | − |
| Collagen alpha-1(XXI) chain | Extracellular matrix organization  Collagen formation  Collagen chain trimerization | − | Down/ -1.89 | − |
| Collagen type I alpha 2 isoform 5 |  | − | Up/ 2.0 | − |
| Collagen alpha-1(III) chain | AGE-RAGE signaling pathway in diabetic complications  Relaxin signaling pathway  Diabetic cardiomyopathy  Protein digestion and absorption | − | Up/ 1.0 | − |
| Collagen alpha-2(I) chain (Alpha-2 type I collagen) |  | − | − | Down/ 1.40 |
| Tyrosine-protein kinase receptor | Modulation of PI3K-Akt-mTOR signaling by bioactive sphingolipids  Ras signaling  FoxO signaling pathway | Up/ 1.55 | − | − |
| Tubulin alpha chain | Amyotrophic lateral sclerosis  Apoptosis  Gap junction | − | Down/ -4.0 | − |
| peptidyl-prolyl cis-trans isomerase |  | Up/ 1.16 | − | − |
| chloride intracellular channel protein 4 |  | Up/ 1.99 | − | − |
| 14-3-3 protein beta/alpha (Protein 1054) | PI3K-Akt signaling pathway  Cell cycle  Hippo signaling pathway | Up/ 1.22 | − | − |
| 40S ribosomal protein S19 | Ribosome  Nervous system development  Cellular responses to stimuli | − | Up/ 1.17 | − |
| Tubulin beta-4B chain | Metabolism of proteins  Cellular responses to stress  Cellular responses to stimuli | Up/ 1.65 | − | − |
| Tubulin beta chain |  | − | − | Up/ 1.76 |
